# Cell-Biological Response and Sub-Toxic Inflammatory Effects of Titanium Dioxide Particles with Defined Polymorphic Phase, Size, and Shape

**DOI:** 10.3390/nano13101621

**Published:** 2023-05-12

**Authors:** Marina Breisch, Mateusz Olejnik, Kateryna Loza, Oleg Prymak, Nina Rosenkranz, Jürgen Bünger, Christina Sengstock, Manfred Köller, Götz Westphal, Matthias Epple

**Affiliations:** 1 BG University Hospital Bergmannsheil, Surgical Research, Ruhr University of Bochum, 44789 Bochum, Germany; 2 Inorganic Chemistry and Center for Nanointegration Duisburg-Essen (CENIDE), University of Duisburg-Essen, 45117 Essen, Germany; 3 Institute for Prevention and Occupational Medicine of the German Social Accident Insurance (IPA), Institute of the Ruhr University of Bochum, 44789 Bochum, Germany

**Keywords:** titanium dioxide, titania, particles, inflammation, macrophages, particle-induced cell migration assay, rutile, anatase

## Abstract

Six types of titanium dioxide particles with defined size, shape, and crystal structure (polymorphic form) were prepared: nanorods (70 × 25 nm^2^), rutile sub-microrods (190 × 40 nm^2^), rutile microspheres (620 nm), anatase nanospheres (100 nm), anatase microspheres (510 nm), and amorphous titania microspheres (620 nm). All particles were characterized by scanning electron microscopy, X-ray powder diffraction, dynamic light scattering, infrared spectroscopy, and UV spectroscopy. The sub-toxic cell-biological response to these particles by NR8383 macrophages was assessed. All particle types were taken up well by the cells. The cytotoxicity and the induction of reactive oxygen species (ROS) were negligible for all particles up to a dose of 100 µg mL^−1^, except for rutile microspheres which had a very rough surface in contrast to anatase and amorphous titania microspheres. The particle-induced cell migration assay (PICMA; based on chemotaxis) of all titanium dioxide particles was comparable to the effect of control silica nanoparticles (50 nm, uncoated, agglomerated) but did not show a trend with respect to particle size, shape, or crystal structure. The coating with carboxymethylcellulose (CMC) had no significant biological effect. However, the rough surface of rutile microspheres clearly induced pro-inflammatory cell reactions that were not predictable by the primary particle size alone.

## 1. Introduction

Titanium dioxide (TiO_2_) occurs in three different polymorphic forms (tetragonal anatase, tetragonal rutile, and orthorhombic brookite), among which anatase and rutile have significant industrial importance. With an annual production of over 3 million tons, TiO_2_ is one of the most important inorganic materials that has been used in many industrial applications for decades [1,2]. Its light-scattering properties caused by a very high refractive index and its semiconducting nature are the reasons for its application as white pigment in varnishes, paints, textiles, foods, and plastics [3,4]. TiO_2_ is almost chemically inert, practically insoluble in aqueous solutions, and non-toxic. In addition, its main application as white pigment, micro- and nanoscale TiO_2_ particles with very small diameters (*d* < 100 nm) are also applied as UV absorber in sunscreens or as additive in food, toothpaste, and pharmaceuticals [5].

The International Agency for Research on Cancer (IARC) has classified titanium dioxide (TiO_2_) as possibly carcinogenic to humans (Group 2B) on the basis of sufficient evidence for carcinogenicity in animal experiments after inhalation. However, the human data were assessed as inadequate for classification [6]. According to German regulatory boards, TiO_2_ is of rather low toxicity. Thus, the German workplace threshold limit (TLV) for granular particles without intrinsic toxicity (GBS) is partly based on TiO_2_ toxicity data. This is based on the assumption that the carcinogenic effects of TiO_2_ are caused by its inflammatory effects at chronic overload of the lung clearance and not by an inherent toxicity. In 2021, the European Union has banned the use of titanium dioxide as food additive (E171) due to health concerns (mainly genotoxicity) caused by ingested nanoparticles [7,8,9,10]. 

The risk assessment for TiO_2_ is complicated by the fact that TiO_2_ is used (and investigated) in different crystalline forms and with many different surface coatings—in particular when it is applied as a pigment in cosmetics, paints or printing inks. A TiO_2_ mixture of 80% anatase and 20% rutile, which is also known as PM25, was mostly used for toxicological assessments [6]. Unfortunately, investigations of TiO_2_ particles with defined crystallographic phase (polymorph) are rare, especially in the sub-toxic range.

Despite its extensive classification as a non-toxic substance, uncontrolled inhalative exposure to TiO_2_ can be hazardous to humans [11,12,13,14]. This has been shown in a number of toxicological studies regarding possible hazards after inhalation [13,14,15,16,17,18]. However, toxicological data in systematic studies to assess TiO_2_ particles depending on their polymorphic phase, their crystallinity, particle size, shape, coating and possible agglomeration behavior are not complete [10,12,13,14,19,20,21,22]. Commercially available TiO_2_ nanoparticles, which usually consist of a rutile–anatase mixture, are often irregular, with a broad particle size distribution and significantly larger hydrodynamic size after redispersion in aqueous media, due to particle agglomeration [23]. This complicates the investigation of the inflammatory and toxicological effects with respect to the particle properties.

Our aim was to close this gap and to synthesize monodisperse and colloidally stable particles by sol-gel chemistry, which enables control over particle size, shape, and crystal form [24,25,26,27]. We pursued various synthetic routes to prepare particles with well-defined properties. Variation parameters were shape (spheres and rods), size (in length or diameter), and crystal structure (i.e., amorphous titania, crystalline anatase and crystalline rutile). Altogether, we prepared six types of TiO_2_ particles. The particles were functionalized with anionic polyelectrolyte carboxymethyl cellulose (CMC) to obtain the same charge in all particle types and to prevent agglomeration and sedimentation as far as possible.

An in-depth cell-biological study with NR8383 macrophages at sub-toxic concentration to compare the effect of these chemically identical TiO_2_ particles was performed to reveal the effect of these parameters on the production of inflammatory markers. The overall goal of this study was a statement as to whether size, shape, and crystal structure of granular bio-persistent dust should be taken into account when setting exposure limit values beyond current legal requirements.

## 2. Materials and Methods

### 2.1. Chemicals 

We used titanium(IV)butoxide (Ti(OBu)_4_, Sigma-Aldrich, St. Louis, MO, USA; 97%), titanium(IV)iso-prop-oxide (Ti(O^i^Pr)_4_, TTIP, Sigma-Aldrich, 97%), titanium tetrachloride (TiCl_4_, Sigma-Aldrich, 99.9%), carboxymethyl cellulose sodium salt (CMC, *M_W_* = 90,000 g mol^−1^, Sigma-Aldrich), calcium chloride dihydrate (CaCl_2_⸱2 H_2_O, Sigma-Aldrich, >99%), methanol (Fisher Chemicals, Schwerte, Germany; p.a.), acetic acid (VWR Chemicals, Darmstadt, Germany, p.a., 99.7%), hydrochloric acid (VWR Chemicals, p.a., 37%), sulfuric acid (Roth, Karlsruhe, Germany; 96%), and potassium hydroxide (Sigma-Aldrich, 90%) as obtained. Ultrapure water was prepared with a Purelab ultra instrument (ELGA LabWater, Celle, Germany). All syntheses and characterizations were carried out with ultrapure water as solvent unless otherwise noted. Prior to the syntheses, all glassware was cleaned with concentrated sulfuric acid (140 °C, 30 min) and thoroughly washed with boiling water. Finally, all glassware was sterilized at 200 °C for 3 h.

### 2.2. Instruments

Dynamic light scattering for particle size analysis and zeta potential determination were carried out with a Malvern Zetasizer Nano ZS ZEN 3600 instrument (Malvern Panalytical Ltd., Malvern, UK; 25 °C, laser wavelength 633 nm; fixed angle 173°; backward scattering mode). A log-normal distribution was assumed as peak profile of the size distribution. The average particle diameter was represented as mean value of the maximum of the size distribution from the log-normal distribution fit. The polydispersity index (PDI) of the system was calculated from DLS measurements. The particles were dispersed in pure water at pH 7.

Ultraviolet-visible spectroscopy (UV/vis) was performed in Suprasil^®^ micro quartz cuvettes with a Varian Cary 300 instrument (Agilent Technologies, Santa Clara, CA, USA). The solvent, as background correction, and the diluted particle dispersion were measured with sample volumes of 750 µL. 

Fourier-transformed infrared spectroscopy (FT-IR) was performed by pressing the samples onto a diamond plate with an Attenuated Total Reflection (ATR) Alpha Platinum FT-IR instrument (Bruker, Billerica, MA, USA).

Combustion analysis was performed with an elemental analyzer EuroVector EA3000 (EuroVector, Pavia, Italy) to quantify the organic matter content in the samples (C, H in CMC).

For X-ray powder diffraction, particle powders that had been dried at 80 °C in air for 4 h were used. X-ray powder diffraction was carried out with a Bruker D8 Advance diffractometer operating in Bragg-Brentano geometry with Cu Kα radiation (*λ* = 1.54 Å, 40 kV, and 40 mA). The samples were placed on single-crystalline silicon sample holders cut to a crystallographic (911) plane to minimize background scattering. The samples were analyzed from 5 to 90° 2Θ with a step size of 0.01° and a counting time of 0.6 s at each step. The instrumental peak broadening was determined with lanthanum hexaboride as internal standard (LaB_6_; NIST, National Institute of Standards and Technology; reference compound). Rietveld refinement was performed with the program package TOPAS 5.0 (Bruker) to determine lattice parameters and fractions of the different crystalline polymorphs of TiO_2_. For the calculation of crystallite size, the Scherrer and Stokes-Wilson equations were used [28]. 

The diffraction pattern of both tetragonal phases of TiO_2_ were taken from the ICDD database (International Centre for Diffraction Data) as reference (anatase: #21-1272, rutile #21-1276) and used for the qualitative phase analysis with Diffrac.Suite EVA V1.2 (Bruker).

Scanning electron microscopy was performed with an Apreo S LoVac instrument (Thermo Fisher Scientific, Waltham, MA, USA) in combination with a Thermo Scientific UltraDry silicon drift X-ray detector on gold/palladium-sputtered samples.

To analyze the cellular uptake of titanium dioxide particles by scanning electron microscopy, the NR8383 cells were rinsed twice with PBS and fixed with a glutaraldehyde solution (3.7% in PBS, Sigma-Aldrich) for 15 min at room temperature. Fixed cells on round glass microscopy slides (Sarstedt, Nümbrecht, Germany) were rinsed twice with PBS again, and then the cells were dehydrated with an ascending sequence of ethanol (20%, 40%, 60%, 80%, 96–98%, 5 min each). Prior to investigation, the samples were sputtered with gold/palladium coating.

### 2.3. Synthesis of TiO_2_ Particles

Amorphous titanium dioxide microspheres were synthesized by a one-pot synthesis according to Han et al. [29]. In a 250 mL round-bottom flask, 200 µL of an aqueous solution of CaCl_2_ (0.05 M) was added to 50 mL methanol. The mixture was stirred for 10 min. Afterwards, 850 µL of TTIP was added dropwise during 1 min and the solution was stirred for 4 h at room temperature. After the reaction was finished, the microspheres were collected by centrifugation (3500 rpm, 30 min, Heraeus Fresco 21 centrifuge Thermo Scientific) and redispersed in water by ultrasonication. After each centrifugation, the supernatant was removed, and the particle pellet was redispersed in water using an ultrasonic bath and dried at 80 °C for 4 h in air.

For the synthesis of anatase microspheres we used the amorphous titanium dioxide microspheres. Dried titanium dioxide microspheres were transferred to a porcelain crucible, heated to 500 °C within one hour and held at that temperature for 1 h. The calcined particles were cooled down to room temperature and then redispersed in water by ultrasonication. The purification of the anatase microspheres was carried out in the same way as for the amorphous particles. 

Anatase nanospheres were synthesized by a solvothermal method according to Ye et al. [30]. In a 50 mL centrifuge tube (Corning, NY, USA), 0.75 mL of Ti(OBu)_4_ was added dropwise to 15 mL acetic acid. After vigorous stirring at room temperature for 20 min, the complete solution was transferred into a 20 mL PTFE-lined autoclave, closed, and annealed at 200 °C for 24 h. After cooling down, the particle solution was collected by centrifugation (3500 rpm, 1 h). The particles were washed three times with water by the same centrifugation procedure and dried at 80 °C for 4 h in air.

The synthesis route for rutile nanorods was adapted and modified from Dong et al. [31]. In a 50 mL centrifuge tube (Corning), 10 mL of Ti(OBu)_4_ was rapidly added under vigorous stirring to an aqueous solution of HCl (30 mL, 3 M) at room temperature. After the complete addition of Ti(OBu)_4_, a solid was formed which completely dissolved within 2 h together with the formation of a two-phase system. After 2 h, the solution was stirred very slowly (50 rpm) for 16 h to allow the formation of the nanoparticles. The rutile nanorods were isolated by centrifugation (3500 rpm, 90 min), washed with water three times by the same centrifugation procedure and dried at 80 °C for 4 h on air.

Rutile sub-microrods were prepared according to Dong et al. [31], with modifications. In a 50 mL centrifuge tube (Corning^®^), 8.5 mL of Ti(OBu)_4_ was added to an aqueous solution of HCl (30 mL, 3 M). The solution was vigorously stirred for 4 h at room temperature. After the stirring was stopped, 15 mL of the aqueous, almost colorless solution (inorganic phase of the formed two-phase system) were carefully removed and transferred to a PTFE vessel for the subsequent hydrothermal treatment. The hydrothermal reaction was carried out in a 20 mL PTFE-lined autoclave at 200 °C for 2 h. After 2 h, the reaction mixture was cooled to room temperature. The purification of rutile sub-microrods was performed by triple centrifugation (3500 rpm, 60 min) and washing with water three times. Finally, the particles were dried at 80 °C for 4 h in air.

The synthesis of rutile microspheres was based on the work of Yan et al. [32] and carried out with some modifications. First, 30 mL water was added into 500 mL three-neck flask under argon and cooled to 0 °C with an ice bath. After 10 min, 3.2 mL of TiCl_4_ was injected with a syringe through a septum in argon atmosphere. Within 20 min, the solution turned from white to almost colorless. Then, 30 mL of an aqueous solution of KOH (1 mol L^−1^, pre-cooled to 0 °C) was added, followed after 5 min by 0.3 mL HCl (37%). After 10 min, 15 mL of the colorless solution was transferred into a 20 mL PTFE-lined autoclave and heated to 100 °C for 24 h. The cooled particle dispersion was purified by centrifugation (3500 rpm, 30 min) and washed with water after redispersion by ultrasonication. The purification steps were repeated 3 times, and finally the rutile microspheres were dried at 80 °C for 4 h in air.

All dried titanium dioxide particles were surface-coated with CMC. In a 50 mL centrifuge tube (Corning^®^), 50 mg of each TiO_2_ type was dispersed in 30 mL water by ultrasonication for 5 min. Then, 10 mL of an aqueous CMC solution (25 g L^−1^) was added, followed by vigorous stirring for 12 h at room temperature. To remove excess CMC, the particles were purified by triple centrifugation in water, as described above. Finally, the CMC-functionalized TiO_2_ particles were dried at 80 °C for 4 h in air.

The number of particles in 1 g solid was computed from the average particle mass for spheres and rods by the following procedure:

$$m_{\mathrm{sphere}}=\frac{4}{3}\;\pi \;r^{3}\;\rho$$

$$m_{\mathrm{rod}}={\pi \;r}^{2}L\;\rho$$

with *r* the particle radius and *L* the particle length, both obtained by SEM analysis (Table 1), and *ρ* the densities of rutile (4200 kg m^−3^), anatase (3900 kg m^−3^), and amorphous TiO_2_ (3650 kg m^−3^) [33,34]. The specific surface area of particles (expressed as m^2^ g^−1^) was calculated as follows:

$$S_{\mathrm{spheres}}=4\pi r^{2}\;N_{\mathrm{particles in 1 g}}$$

$$S_{\mathrm{rods}}=(2\pi r^{2}+2\pi rL)\;N_{\mathrm{particles in 1 g}}$$

### 2.4. Particle Storage and Treatment 

After synthesis, the TiO_2_ particles were stored at 4 °C in the dark under argon atmosphere. Stock solutions in sterile water were adjusted to a particle concentration of 4 mg mL^−1^. Immediately before use in cell culture experiments, the particles were dispersed by ultrasonication (Elma Sonic S10, Elma Ultrasonic, Singen, Germany; 30 W, 5 min) and thorough vortexing. They were applied to the cells at the final concentrations in a well of 25, 50, 100, 200, and 300 µg mL^−1^, respectively.

### 2.5. Cell Culture 

To study the biological activity of the TiO_2_ particles, the rat alveolar macrophage cell line NR8383 (LGC Standards GmbH, Wesel, Germany) was used. Cultivation of cells was carried out in Ham’s F-12 medium with additional 15% fetal calf serum (FCS, GIBCO, Invitrogen, Karlsruhe, Germany) at standard cell culture conditions (humidified atmosphere, 37 °C, 5% CO_2_) in 175 cm^2^ cell culture flasks (BD Falcon, Becton Dickinson GmbH, Heidelberg, Germany). The partly adherent NR8383 cells (1:1 ratio of adherent:non-adherent cells) were scraped from cell culture flasks with a cell scraper (TPP Techno Plastic Products AG, Trasadingen, Switzerland) and mixed with the non-adherent cell portion before seeding 2.4 × 10^5^ cells cm^−2^ in 24-well cell culture plates (BD Falcon; Fisher Scientific).

### 2.6. Cytotoxicity Assay 

The toxic effects of the different TiO_2_ particles at various concentrations (25–300 µg mL^−1^) on NR8383 cells compared to unexposed control cells were evaluated by propidium iodide staining (PI, Sigma-Aldrich) of non-viable cells and flow cytometry. Briefly, after particle exposure for 2 h or 16 h, cells were collected as described above and transferred to 5 mL tubes (BD Biosciences). PI staining was performed with 50 µg mL^−1^ PI for 10 min at RT in the dark. Subsequently, the fraction of non-viable (PI positive) cells was detected by flow cytometry (FACSCalibur, BD Bioscience, Heidelberg, Germany) and quantified with the CELLQuest 1.2.2 software (BD Biosciences). A total number of 15,000 cells was analyzed for each measurement (see Figure S1 for details). 

### 2.7. Generation of Reactive Oxygen Species (ROS)

The intracellular generation of ROS in NR8383 cells upon incubation with the different TiO_2_ particles (25–300 µg mL^−1^) was measured by the DCF assay. After 2 h of particle exposure, the cells were collected and treated with the cell-permeable ROS indicator 2′,7′-dichlorodihydrofluorescein diacetate (20 µM H_2_DCFDA, Thermo Fisher Scientific, Waltham, USA) for 30 min at 37 °C in the dark. In addition, the cells were incubated for 30 min with a hydrogen peroxide solution (3% H_2_O_2,_ Herbeta Arzneimittel, Berlin, Germany), which served as a positive control for enhanced ROS levels. The conversion of the non-fluorescent H_2_DCFDA dye into the highly fluorescent 2′,7′-dichlorofluorescein (DCF) form by generated ROS was quantified by flow cytometry (15,000 cells per measurement). Additional PI staining allowed for discrimination between viable and non-viable cells (see Figure S2 for details). 

### 2.8. Protein Microarray 

Microarray analysis was performed with supernatants of NR8383 cells after 16 h of exposure to 100 µg mL^−1^ of the different TiO_2_ particles with a Proteome Profiler Rat XL Cytokine Array (Bio-Techne GmbH, Wiesbaden-Nordenstadt, Germany). This nitrocellulose membrane-based sandwich immuno-array allowed a semi-quantitative analysis of 79 different bioactive factors (e.g., cytokines, growth factors). The supernatants of particle-treated NR8383 cells were centrifuged at 300 g for 10 min and stored at −20 °C. Subsequent microarray analysis was carried out according to the manufacturer’s instructions. Briefly, supernatants were applied to the membranes, where captured antibodies, spotted as duplicated dots, bound the target proteins from the sample. The captured proteins were then detected with biotinylated detection antibodies, followed by visualization with chemiluminescent detection reagents. Detection and quantification of the chemiluminescence signals were performed with an Amersham Imager 600 RGB and the ImageQuantTL software (Amersham, GE Healthcare Bio Science, Uppsala, Sweden). According to the proteomic repertoire of NR8383 [35], 27 factors were considered for detailed review.

### 2.9. Particle Induced Cell Migration Assay (PICMA) 

PICMA was performed as described earlier [23]. Briefly, NR8383 rat macrophages (LGC Standards, Wesel, Germany) were cultivated at 37 °C, 100% humidity, and 5% CO_2_ in Ham’s F-12 + 15% FCS (PAN-Biotech GmbH, Aidenbach, Germany), 2 mM L-glutamine, 100 µg mL^−1^ penicillin, and 100 U mL^−1^ streptomycin (PAN-Biotech GmbH, Aidenbach, Germany). Approximately 3 × 10^6^ cells were seeded in 25 mL (175 cm^2^) medium. 

We used trans-retinal differentiated HL-60 cells (dHL-60) for the investigation of chemotaxis. The HL-60 cells (DSMZ, Braunschweig, Germany) were differentiated in RPMI 1640 medium +10% FCS, 2 mM L-glutamine, 100 µg mL^−1^ penicillin, 100 U mL^−1^ streptomycin and 1 μM trans-retinoic acid (Sigma-Aldrich, Steinheim, Germany) for three days. 

For particle incubation, 3 × 10^6^ NR8383 cells mL^−1^ were seeded in 12.5 cm^2^ cell culture flasks at a final volume of 3 mL (2.4 × 10^5^ cells cm^−2^). A sample in which cells without particles were incubated served as negative control. Incubation of the NR8383 cells with the particles was performed for 16 h. Thereafter, the cells were removed by centrifugation at 300 g for 5 min, and the particles were removed by centrifugation with 15,000 g for 10 min at room temperature. 

Cell migration was investigated as introduced by Boyden [36], with modifications described by Westphal et al. [37]. Briefly, 200,000 dHL-60 cells were cultured in 200 µL RPMI 1640 medium without FCS and seeded in each plate. A well insert (THINCERT, 3 µm pore size, Greiner bio-one, Frickenhausen, Germany) was put into the cavities of black 24-well plates (Krystal, Dunn Labortechnik, Asbach, Germany) and then 500 µL of the supernatants of the NR8383 cells treated with TiO_2_ particles were added to the lower chamber. Migration of dHL-60 cells was performed for 24 h. 0 to 100,000 HL-60 cells were seeded directly into four-well plates that were left without inserts for calibration. Migrated cells and calibration cells were stained with calcein (Sigma-Aldrich). The cell count was determined by fluorescence spectroscopy at 490/520 nm (SpectraMax M3, Molecular Devices, Sunnyvale, CA, USA). As reference, we used uncoated silica nano particles (CAS No. 7631-86-9, Lot MKBF2889V, 99.5%, 10–20 nm) (Sigma-Aldrich) [23]. 

### 2.10. Statistical Analysis

Data are expressed as the mean ± SD and given as the percentage of the control (cells not exposed to particles). For statistical evaluation, one-way analysis of variance (ANOVA) with Dunett’s Multiple Comparison Test was applied with the GraphPad Prism software (GraphPad Software Inc., San Diego, CA, USA). *p* values ≤0.05 were considered as statistically significant.

## 3. Results

Six different types of titanium dioxide particles with defined shape (spheres, rods), particle size, and crystal structure (anatase, rutile or amorphous), all both unfunctionalized (“naked”) and CMC-coated, were wet-chemically prepared. The particles were comprehensively characterized by different analytical methods, i.e., scanning electron microscopy (SEM), dynamic light scattering (DLS), UV/vis spectroscopy, infrared (IR) spectroscopy, X-ray powder diffraction (XRD), and elemental analysis (EA). All characterization results are summarized in Table 1. The content of CMC is approximately two times the carbon content (as carbon could also come from impurities), i.e., the particles contained between 1 and 5 wt% CMC.

**Figure 1:**
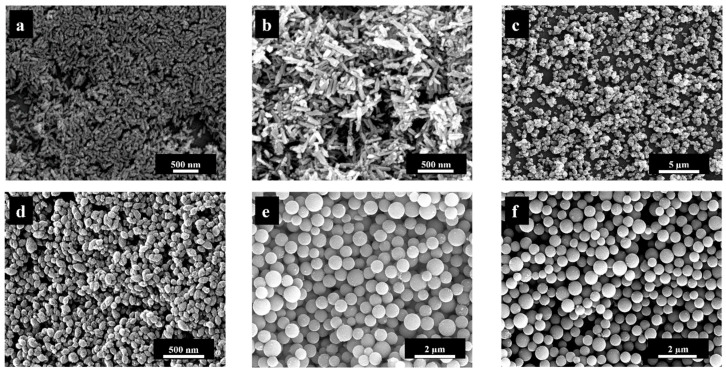
Representative SEM images of unfunctionalized titanium dioxide particles: Rutile nanorods (**a**), rutile sub-microrods (**b**), rutile microspheres (**c**), anatase nanospheres (**d**), amorphous _titania_ microspheres (**e**), and anatase microspheres (**f**).

Particle size and shape of all titanium dioxide particles were determined by SEM. Scanning electron micrographs showed that the control of the particle shape (spherical or rod-shaped) and the control of the particle size in three ranges (“nano”, “sub-micro”, “micro”) were made possible by variation of the reaction parameters. Another challenge was the control of the polymorphic phase, i.e., anatase or rutile. Figure 1 shows scanning electron micrographs of all particles. There was no difference between unfunctionalized and CMC-coated particles, except for very high-resolution images, where the polymer led to a slightly blurred particle surface.

**Figure 2:**
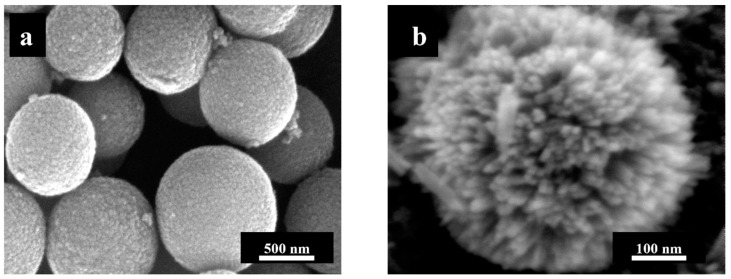
High-resolution SEM images of the surface of unfunctionalized titanium dioxide particles: Anatase microspheres (**a**) and rutile microspheres (**b**).

As can be seen in Figure 2, the anatase microspheres consisted of spherical crystallites and had a very smooth surface, while the rutile microspheres were significantly rougher. This is visible on the particle surface, which consisted of stacked nanorods. The surface of the amorphous titania microspheres was also very smooth (see Figure 1e).

**Figure 3:**
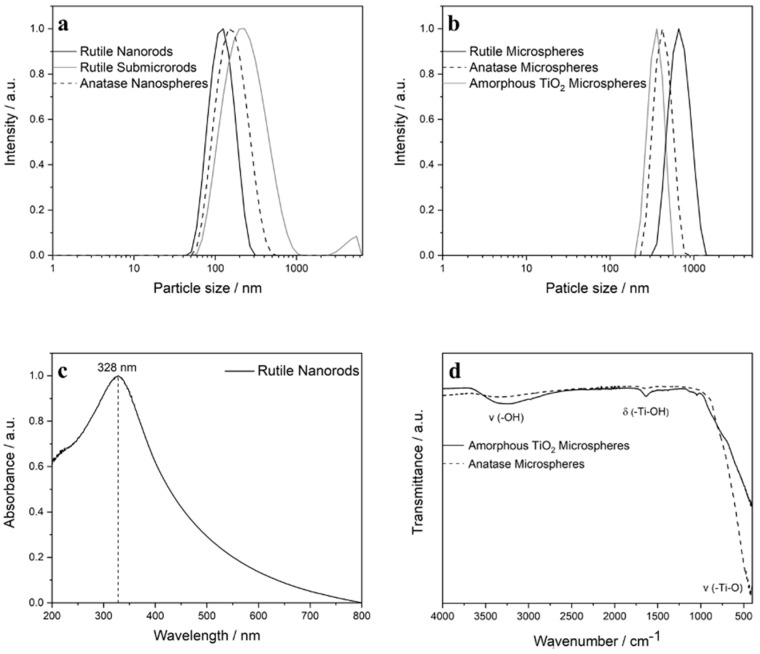
Representative characterization data of unfunctionalized TiO_2_ particles: DLS data of TiO_2_ nano- and sub-micro particles (**a**), DLS data of TiO_2_ microparticles (**b**), UV/vis spectroscopy of rutile nanorods (**c**), and IR spectroscopy of amorphous titania microspheres before calcination and anatase microspheres after calcination (**d**). The other particle shapes gave similar results.

Dynamic light scattering (DLS) was used to investigate the colloidal stability and the particle size distribution in aqueous dispersion (Figure 3). In all cases the polydispersity index (PDI) was below 0.3, which indicated a good dispersibility. The dispersed particles were not strongly agglomerated, as the comparison between the diameters by SEM and by DLS indicates (Table 1). 

The zeta potential was also determined by dynamic light scattering. Due to the presence of Ti-OH groups on the particle surface, the unfunctionalized TiO_2_ particles were electrostatically stabilized, as indicated by the zeta potential. The coating of the particles with the anionic polyelectrolyte carboxymethylcellulose (CMC) led to charge reversal and an electro-steric stabilization.

In addition to the zeta potential measurements, the particles were analyzed by IR spectroscopy (Figure 3). IR spectra of amorphous microspheres and anatase microspheres showed characteristic vibrations of Ti-O-Ti bonds at 403 cm^−1^ and of Ti-OH groups at 1636 cm^−1^ [38], whose intensity decreased after calcination due to condensation under release of water (additional IR spectra are shown in Figures S12–S17). UV-vis spectroscopy provided information about the optical properties of the particles (Figure 3). As expected, only the rutile nanorods gave a near-UV absorption peak at 328 nm [39]. Additional UV spectra are shown in Figures S18–S21.

**Table 1:** Physicochemical characterization data of unfunctionalized and CMC-coated TiO_2_ particles. Standard deviation of the last digits given in parentheses.

	Size by SEM/nm	Size by DLS/nm	PDI from DLS	Zeta Potential by DLS/mV	Size by DLS/nm	PDI from DLS	Zeta Potential by DLS/mV	Carbon Content by EA/wt%
		**Unfunctionalized (“Naked”)**			**CMC-Coated**			
**Rutile particles**								
Nanorods	70 (20) × 25 (6)	106	0.12	+38	233	0.23	−17	1.8
Sub-microrods	190 (60) × 40 (10)	207	0.24	+25	209	0.27	−24	0.5
Microspheres	620 (160)	509	0.19	+23	650	0.38	−6	0.4
**Anatase particles**								
Nanospheres	100 (21)	143	0.05	+33	161	0.14	−20	0.6
Microspheres	510 (89)	618	0.05	+23	574	0.11	−29	1.5
**Amorphous particles**								
Microspheres	620 (101)	649	0.10	−25	705	0.03	−29	2.5

**Figure 4:**
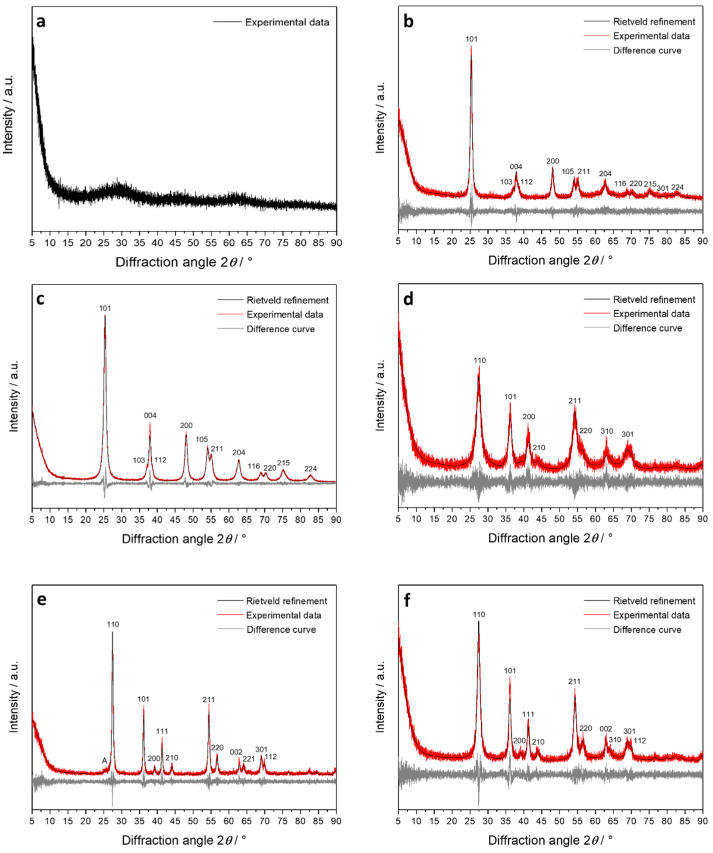
X-ray powder diffractograms of TiO_2_ particles: Amorphous titania microspheres (**a**), anatase microspheres (**b**), anatase nanospheres (**c**), rutile nanorods (**d**), rutile sub-microrods, containing a small amount of anatase (A) (**e**), and rutile microspheres (**f**).

The TiO_2_ samples were examined by X-ray powder diffraction to assess their crystal structure, followed by quantitative Rietveld refinement (Figure 4 and Table 2). TiO_2_ microspheres prepared from titanium alcoholates by sol-gel chemistry were fully X-ray amorphous. The phase transition from amorphous to crystalline during the calcination step led to the formation of pure anatase. Anatase nanospheres, rutile nanorods, and rutile sub-microrods were phase-pure, probably determined by the type of acid used during the synthesis. The rutile sub-microrods contained a small impurity of anatase. The Rietveld refinement data of the crystalline TiO_2_ particles agreed well with the literature data for both polymorphs, i.e., anatase (tetragonal, space group *I*4_1_/amd with *a* = 3.785 Å and *c* = 9.514 Å) and rutile (tetragonal, space group *P*4_2_/mnm with *a* = 4.593 Å and *c* = 2.959 Å). The mean crystallite size of all crystalline particles from Rietveld refinement was smaller than the particle size observed by SEM (see Table 1), indicating a polycrystalline nature of all particles. In other words, each particle shown in SEM consists of a number of smaller crystallites, i.e., it is not a single crystal.

**Figure 5:**
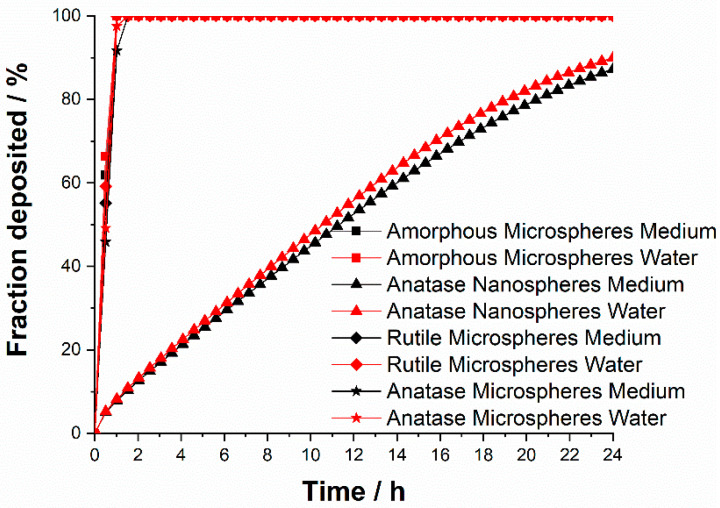
Precipitation and diffusion of TiO_2_ particles according to the ISDD model [42] in water and in cell culture medium (DMEM supplemented with 10% FCS).

Particle precipitation and diffusion in cell culture medium depend on particle size, agglomeration state, density, and surface coating. This can significantly affect the dose which an adherent cell experiences at the bottom of a cell culture dish. For that reason, we have estimated the particle transport in water and in cell culture media (DMEM supplemented with 10% FCS) by simultaneous solution of the Stokes-Einstein equation and Stokes Law, with the in vitro sedimentation, diffusion and dosimetry model (ISDD) [40,41,42]. Figure 5 shows precipitation and diffusion rates of TiO_2_ particles according to the ISDD model in water and in cell culture medium. The fast precipitation of TiO_2_ microspheres is due to sedimentation, whereas the slower transport of nanoparticles is affected by diffusion and sedimentation. There was no significant difference between water and cell culture medium as dispersion medium.

**Figure 6:**
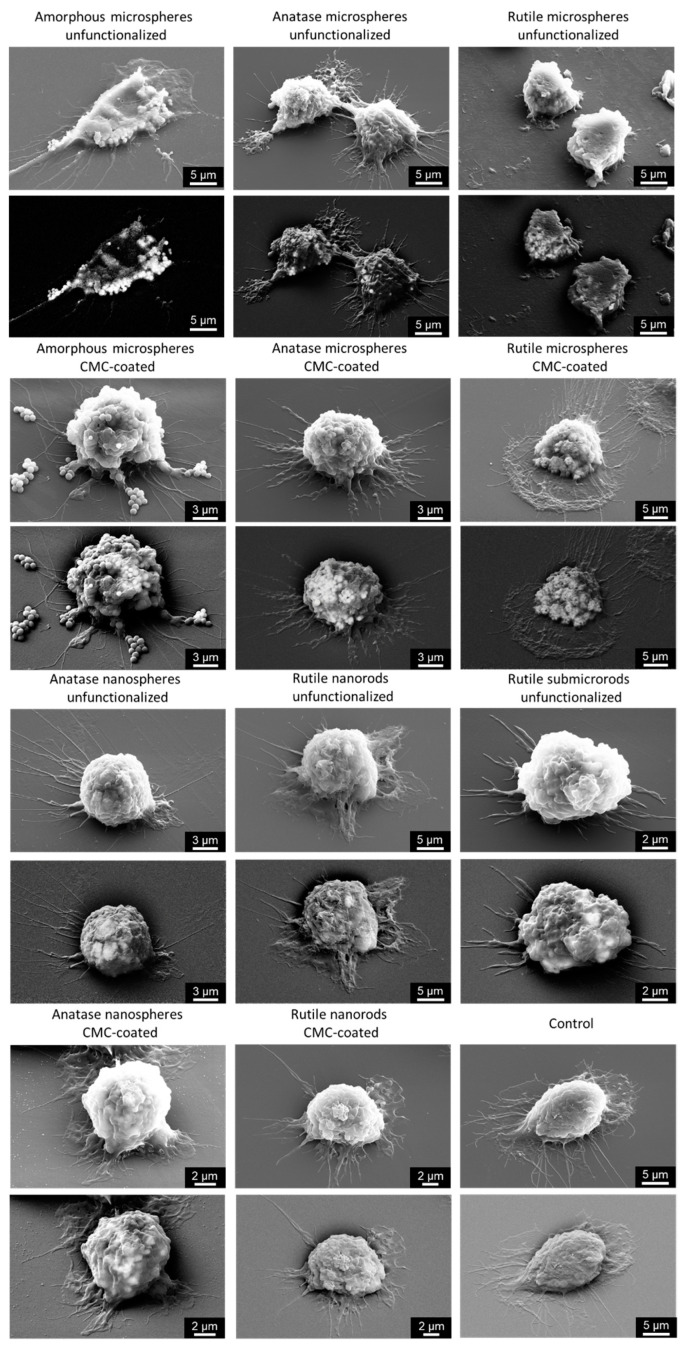
Representative scanning electron micrographs of NR8383 cells after 24 h of incubation with 50 µg mL^−1^ TiO_2_ particles with secondary electron detector (top image for each sample) and backscattering electron detector (bottom image for each sample).

The uptake of TiO_2_ particles by NR8383 cells after 24 h was assessed by backscattered electron (BSE) imaging in a scanning electron microscope, taking advantage of the dependence of the contrast from the atomic number *Z*. Titanium (atomic number *Z* = 22) appears brighter in a BSE image compared to lighter elements, such as carbon (atomic number *Z* = 6), nitrogen (atomic number *Z* = 7), or oxygen (atomic number *Z* = 8). All particles were strongly taken up by the cells, irrespective of their size, shape or CMC coating, well in line with general observations of the interaction of nanoparticles [43,44,45,46,47] and microparticles [47,48,49,50] with cells (Figure 6). 

**Figure 7:**
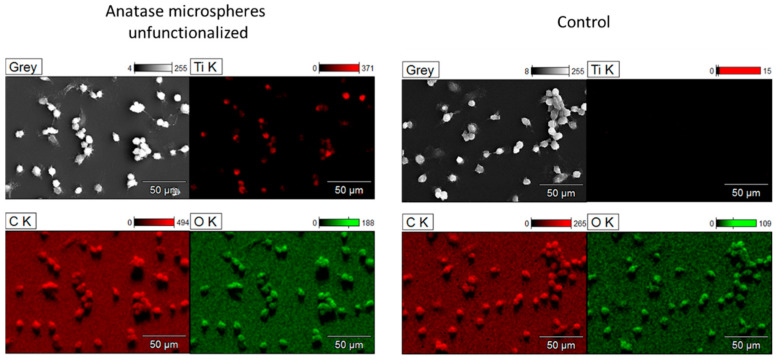
EDS elemental mapping of NR8383 macrophages after incubation with TiO_2_ particles for 24 h (unfunctionalized anatase microspheres; **left**) and untreated cells for comparison (**right**). EDS data of the other particle types can be found in Figures S3–S11.

Energy-dispersive X-ray spectroscopy (EDS) was performed to confirm the particle uptake by NR8383 macrophages and the particle identity. Figure 7 shows representative EDS elemental mapping for untreated cells and after incubation with anatase microspheres. The SEM and EDS images clearly demonstrate that all types of TiO_2_ particles were taken up well by the cells. However, a quantitative evaluation of the uptake is not possible from these data.

**Figure 8:**
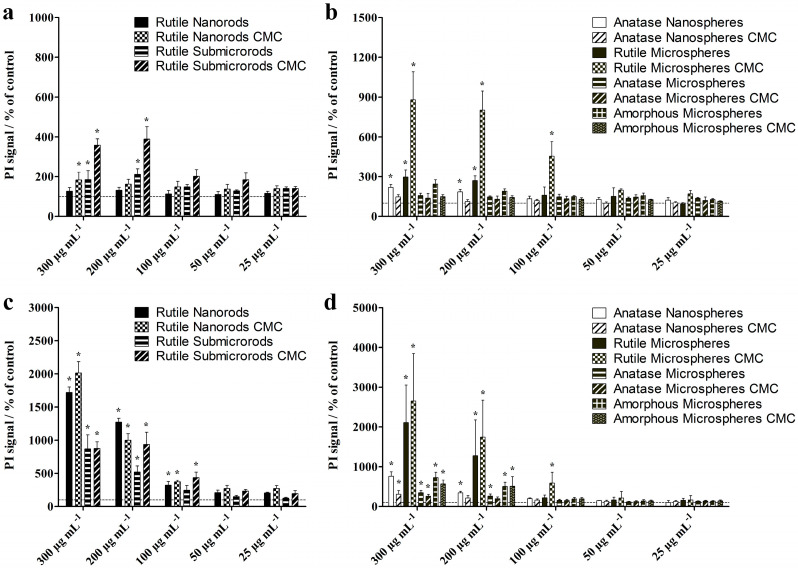
Toxic effects on NR8383 alveolar macrophages after 2 h (**a**,**b**) and 16 h (**c**,**d**) of exposure to TiO_2_ rods (**a**,**c**) and TiO_2_ spheres (**b**,**d**) at different concentrations. The cell viability was assessed by staining of non-viable cells with PI and flow cytometric analysis of the mean PI fluorescence intensity. Data are expressed as mean ± SD (*n* = 3), given as percentage of control (100%, non-treated cells). Asterisks (*) indicate significant differences in comparison to the control (* *p* ≤ 0.05).

Potential cytotoxic effects of the different TiO_2_ particles on NR8383 alveolar macrophages were determined by flow cytometric analysis of PI-labeled, non-viable cells after 2 h and 16 h of incubation (Figure 8). After 2 h of incubation, all rutile particles (nanorods, sub-microrods, microspheres) and anatase nanospheres induced a significant cytotoxicity compared to non-treated control cells, although only at high particle concentrations of ≥100 µg mL^−1^. For all types of TiO_2_ particles, the cell toxicity increased with prolonged incubation time, which was reflected by the increased number of PI-positive cells. However, even after 16 h of exposure, none of the particles exhibited significant effects on the cell viability at concentrations below 100 µg mL^−1^. Among all tested particles, rutile microspheres had the highest cytotoxic activity, which was possibly caused by the rough particle surface consisting of stacked nanorods (see Figure 2b). The CMC-coating had no statistically significant effect on the biological activity of anatase and amorphous spheres, but in the case of rutile particles, most apparently for microspheres, unfunctionalized particles were less toxic than CMC-functionalized ones.

**Figure 9:**
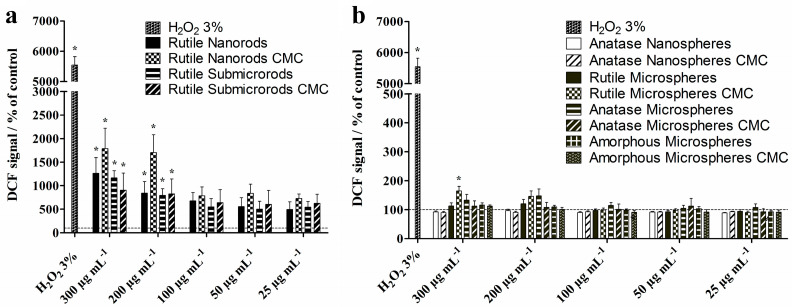
ROS levels in NR8383 alveolar macrophages after 2 h of exposure to TiO_2_ rods (**a**) and TiO_2_ spheres (**b**) at different concentration. Generation of ROS was determined by flow cytometric analysis of the mean fluorescence intensity of dichloro-fluorescein (DCF). A 3% H_2_O_2_ solution served as positive control for elevated ROS levels. Data are expressed as mean ± SD (*n* = 3), given as the percentage of control (100%, non-treated cells). Asterisks (*) indicate significant differences in comparison to the control (* *p* ≤ 0.05).

The formation of reactive oxygen species (ROS) in NR8383 macrophages after 2 h of incubation with the different TiO_2_ particles was examined by the DCF assay and flow cytometry (Figure 9). Compared to non-treated control cells, NR8383 cells exposed to 200 and 300 µg mL^−1^ rutile nano- and sub-microrods caused significantly enhanced ROS levels, while, in the presence of the spherical particles, only a minor effect on ROS generation was detected. Overall, at moderate particle concentrations, none of the TiO_2_ particles provoked cell activation associated with elevated ROS. 

**Figure 10:**
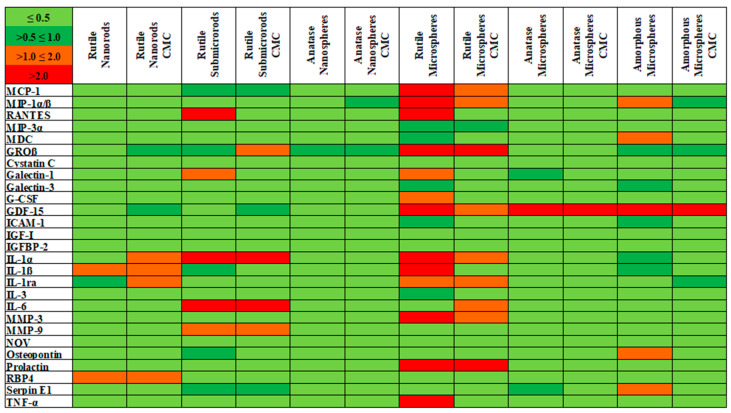
Heat map of selected bioactive factors expressed by NR8383 alveolar macrophages after 16 h of exposure to different TiO_2_ particles (100 µg mL^−1^). The expression of 27 factors, selected according to the proteomic NR8383 repertoire, was identified by a protein microarray. The standardized expression of the individual factors is presented relative to the control (i.e., no particle exposure) and encoded by the color intensity scale in the upper left corner (green for low expression, red for high expression).

To further analyze possible cell-activating effects of the TiO_2_ particles, the expression of bioactive factors was detected from NR8383 cell supernatants after 16 h of exposure to 100 µg mL^−1^ of the different particles. 79 bioactive molecules, including cytokines, chemokines, and growth factors, were detected simultaneously from each cell supernatant sample with a protein microarray. According to the proteomic repertoire of the NR8383 cell line [35], 27 factors were selected for a detailed view and are presented in the qualitative heat map in Figure 10. 

An increase in the relative expression levels of the selected bioactive factors was observed for rutile sub-microrods, and this was even stronger for rutile microspheres, as indicated by an overall higher red content in the heat map. Rutile nanorods and anatase and amorphous spheres, by contrast, showed a higher green content, which indicated a reduced expression. Furthermore, an increased expression of GDF-15 was detected for all microspheres. GDF-15 expression was also observed earlier after challenge of NR8383 cells with asbestos fibers and multiwalled carbon nanotubes (MWCNT) [37]. It should be noted that the protein array analysis represents a qualitative screening method that provides a first overall impression of possible increase/decrease in expression patterns. However, a subsequent quantitative ELISA analysis revealed no statistically significant size-, morphology-, or structure-related effects and, therefore, no cell activation for the examined TiO_2_ particles. This confirms that the exposure to all TiO_2_ particles was in the sub-toxic region.

**Figure 11:**
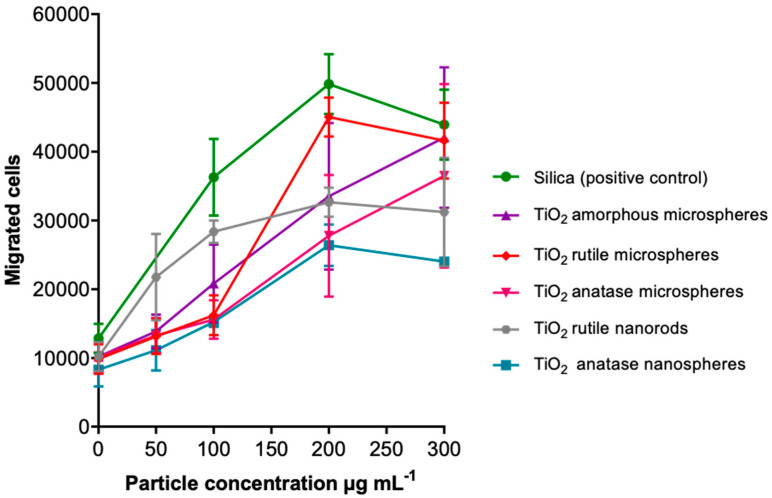
PICMA assay: Chemotaxis (migrated cells) of the unexposed dHL-60 cells in response to NR8383 cell supernatants that were obtained from incubation with TiO_2_ particle concentrations between 50 and 300 µg mL^−1^. Data are expressed as mean ± SD (*n* = 3). Commercial silica nanoparticles served as positive control.

Following our approach to elucidating the pro-inflammatory potential at sub-toxic concentrations, the attraction of dHL60 cells by supernatants from particle-treated NR8383 cells was investigated in the examined particles as an in vitro estimate for neutrophilic inflammation (Figure 11). The effects of all TiO_2_ particles were smaller compared to the silica positive control. The TiO_2_ curves were scattered around that of amorphous TiO_2,_ which showed a linear slope. Rutile nanorods showed the steepest slope and a hyperbolic course, whereas rutile microspheres exhibited the highest peak but a sigmoidal curve shape. Anatase micro- and nanospheres showed the weakest effects and nearly linear courses. Thus, the different TiO_2_ particles caused different cell migration effects that were below the effect of the silica control particles (positive control; 50 nm, strongly agglomerated [23]) but not correlated with specific particle characteristics.

Table 3 summarizes the particle concentration data at the subtoxic concentration of 100 µg mL^−1^. Wiemann et al. [51] have defined a threshold of 4000 µm^2^ particle surface area per cell for a non-toxic interaction with cells. As we are in all cases at or above this limit at a dose of 100 µg mL^−1^, we can conclude that the effect of the particles was not due to a specific cytotoxicity but rather to an unspecific overload of the cells by non-soluble and inherently non-toxic particles. This confirms the observation that TiO_2_ particles do not have an inherent toxicity.

Rutile microspheres had a particularly rough surface (Figure 2), i.e., the actual surface area was much higher than assessed by the particle geometry, which may explain its comparatively high pro-inflammatory activity. This is corroborated by the fact that anatase and titania microspheres of the same shape and almost the same size, but with a smooth surface, did not induce adverse reactions to cells.

We have earlier reported PICMA results for barium sulphate particles [52], for silica particles [53], for silica fibers [54], and for zinc oxide particles [55], all recorded in the sub-toxic range. The results reported here are in good agreement with these findings and confirm the suitability of PICMA to assess the pro-inflammatory effect of micro- and nanoparticles. Barium sulfate as insoluble and bioinert material did not induce cell migration or proinflammatory effects [52]. Silica nanoparticles of different size and shape [53] and silica microrods [54] all had a low pro-inflammatory potential, possibly due to a hydrated surface in comparison to strongly pro-inflammatory quartz [23]. Zinc oxide as partially soluble material had a moderate pro-inflammatory effect that was attributed to the release of zinc ions [55]. Thus, PICMA is able to predict pro-inflammatory effects for particles at the sub-toxic level. This is important for its application as a rapid test for particles on the nano- and on the microscale. Clearly, the assessment of the acute toxicity is not sufficient to elucidate the inflammatory potential associated with particles of all kinds. The results presented here for titanium dioxide as one of the most prominent and most intensely discussed nano-material clearly underscore this fact.

## 4. Conclusions

The in-vitro cell culture investigations showed no influence of size, shape, and crystal structure of chemically identical, granular TiO_2_ particles on the investigated biological effects. Similar small differences of the biological effects that were not linked to distinct particle characteristics were earlier observed for zinc oxide particles [55] and silica particles [53]. Neither the particle-induced cell migration assay (PICMA), the cell toxicity, apoptosis, the release of signaling molecules, nor the formation of ROS showed effects that were correlated with the particle characteristics. The only major exception was the rough surface of rutile microspheres which—in contrast to the smooth surface of anatase microspheres and amorphous titania microspheres—induced pro-inflammatory effects. The dispersion state of the particles and their charge as changed by the application of a shell of carboxymethylcellulose (CMC) did not influence the cell-biological results. Taken together, these results suggest that shape and size of granular bio-persistent dust do not need to be considered for the setting of exposure limit beyond current regulatory provisions. However, comparative studies on other granular particles are desirable to elucidate whether these results can be generalized beyond titania.

## Figures and Tables

**Table 2:** X-ray powder diffraction data of crystalline TiO_2_ particles (unfunctionalized) by Rietveld refinement. *a* and *c* lattice parameters of the tetragonal unit cell; *ρ* X-ray density; *CS* crystallite size. *: The rutile sub-microrods contained 96.4 wt% rutile and 3.6 wt% anatase.

	Rutile Nanorods	Rutile Sub-Microrods *	Rutile Microspheres	Anatase Nanospheres	Anatase Microspheres
*a*/Å	4.620 (1)	4.605 (1)	4.610 (6)	3.794 (3)	3.791 (3)
*c*/Å	2.960 (2)	2.963 (2)	2.959 (4)	9.514 (7)	9.507 (8)
*ρ*/g cm^−3^	4.19	4.22	4.22	3.87	3.88
CS/nm	6.2 (1)	25 (1)	10.3 (10)	11.5 (10)	15 (1)

**Table 3:** Concentration and dose data for all cell culture studies with TiO_2_ particles (100 µg mL^−1^) in a 24-well plate (2 cm^2^, 640 µL, 48,000 cells). As the cytotoxic effects of particles are related to their total surface area, and also with respect to the number of cells, the data were converted to different ratios (see discussion). The particle numbers and the specific surface area were computed from the SEM particle size data.

	TiO_2_ Concentration/mmol L^−1^	TiO_2_ Particle Concentration/L^−1^	TiO_2_ Particle Surface Area/µm^2^ Per Particle	Total TiO_2_ Particle Surface Area/µm^2^ L^−1^	Total TiO_2_ Particle Surface Area Per Well/µm^2^	Total TiO_2_ Particle Surface Area Per Cell/µm^2^
Rutile nanorods	1.25	6.74 × 10^14^	6.15 × 10^−3^	4.1 × 10^12^	2.6 × 10^9^	55,200
Rutile sub-microrods	1.25	1.07 × 10^14^	0.241	2.6 × 10^12^	1.6 × 10^9^	34,300
Rutile microspheres	1.25	1.93 × 10^11^	1.20	0.2 × 10^12^	1.5 × 10^8^	3100
Anatase nanospheres	1.25	4.90 × 10^13^	3.14 × 10^−2^	1.5 × 10^12^	1.0 × 10^9^	21,000
Anatase microspheres	1.25	3.61 × 10^11^	0.83	0.3 × 10^12^	1.9 × 10^8^	4000
Amorphous microspheres	1.25	2.21 × 10^11^	1.20	0.3 × 10^12^	1.7 × 10^8^	3500

## Data Availability

The data presented in this study are available on request from the corresponding author.

## Supplementary Materials

The following supporting information can be downloaded at: https://www.mdpi.com/article/10.3390/nano13101621/s1. In the supplementary data, IR and UV spectra of all particles, further EDS maps, and gating procedures for the analysis of flow cytometry data can be found. Figure S1: Gating strategy for the detection of non-viable NR8383 cells by flow cytometric analysis using the example of unexposed cell control (a), rutile nanorods (b) und CMC-functionalized rutil nanorods (c). Figure S2: Gating strategy for the detection of generated ROS in NR8383 cells by flow cytometric analysis using the example of cells exposed to 3% H_2_O_2_ for 2 h. Figure S3: EDS elemental mapping of NR8383 macrophages after incubation with CMC-functionalized anatase microspheres for 24 h. Figure S4: EDS elemental mapping of NR8383 macrophages after incubation with unfunctionalized anatase nanospheres for 24 h. Figure S5: EDS elemental mapping of NR8383 macrophages after incubation with CMC-functionalized anatase nanospheres for 24 h. Figure S6: EDS elemental mapping of NR8383 macrophages after incubation with unfunctionalized rutile microspheres for 24 h. Figure S7: EDS elemental mapping of NR8383 macrophages after incubation with CMC-functionalized rutile microspheres for 24 h. Figure S8: EDS elemental mapping of NR8383 macrophages after incubation with unfunctionalized rutile submicrorods for 24 h. Figure S9: EDS elemental mapping of NR8383 macrophages after incubation with CMC-functionalized rutile submicrorods for 24 h. Figure S10: EDS elemental mapping of NR8383 macrophages after incubation with unfunctionalized rutile nanorods for 24 h. Figure S11: EDS elemental mapping of NR8383 macrophages after incubation with CMC-functionalized rutile nanorods for 24 h. Figure S12: IR spectrum of amorphous TiO_2_ microspheres. Figure S13: IR spectrum of anatase microspheres. Figure S14: IR spectrum of anatase nanospheres. Figure S15: IR spectrum of rutile nanorods. Figure S16: IR spectrum of rutile submicrorods. Figure S17: IR spectrum of rutile microrods. Figure S18: UV/vis spectrum of amorphous TiO_2_ microspheres. Figure S19: UV/vis spectrum of anatase nanospheres. Figure S20: UV/vis spectrum of rutile nanorods. Figure S21: UV/vis spectrum of rutile submicrorods.
