# Supplementary material for: Cell-Biological Response and Sub-Toxic Inflammatory Effects of Titanium Dioxide Particles with Defined Polymorphic Phase, Size, and Shape
Source: Nanomaterials (Basel). 2023 May 12;13(10):1621. doi: 10.3390/nano13101621 (PMC10221891; doi:10.3390/nano13101621)
Supplement: Supplementary file 1 [file nanomaterials-13-01621-s001.zip › nanomaterials-2353361-supplementary.pdf]

Supplementary Information

Cell-biological response and sub-toxic inflammatory effects of titanium dioxide particles with defined polymorphic phase, size, and shape

Marina Breisch, Mateusz Olejnik, Kateryna Loza, Oleg Prymak, Nina Rosenkranz, Jürgen Bünger, Christina Sengstock, Manfred Koeller, Götz Westphal, Matthias Epple

Cytotoxicity assay FACS analysis

The toxic effects of the different TiO_2 particles at various concentrations on NR8383 cells were evaluated by propidium iodide staining of non-viable cells and flow cytometry. A total number of 15,000 cells were analyzed for each measurement. The gating strategy for the detection of non-viable cells is illustrated in Figure S1. First, the cell fraction was gated to exclude apparent cell debris. The cell gate (=NR8383) was then applied to all events and a second gate was set to the PI-positive fraction which corresponds to the fraction of dead cells (given as mean±SD; n=3) and percentage of control (100%, non-treated cells).

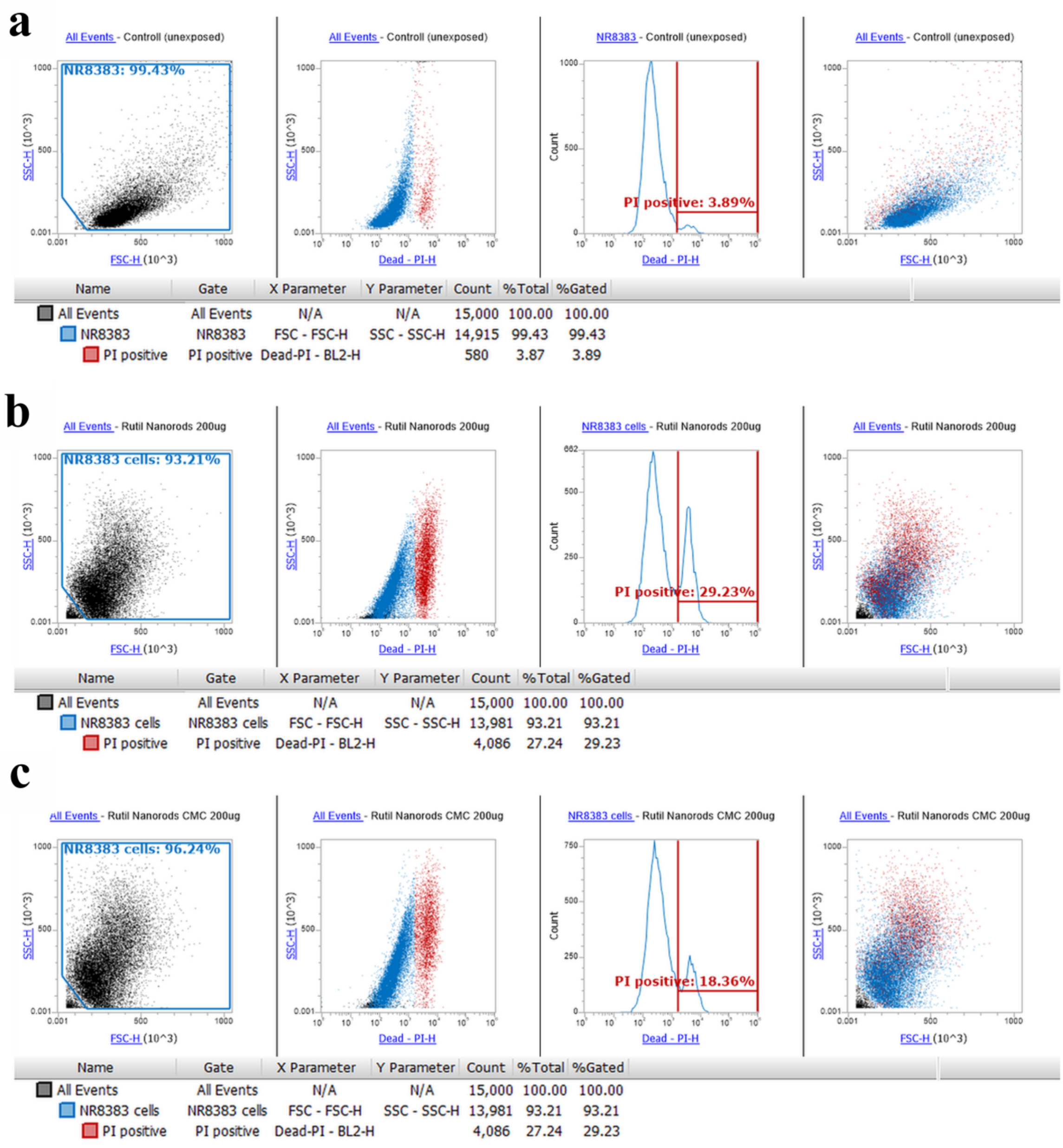

Figure S1: Gating strategy for the detection of non-viable NR8383 cells by flow cytometric analysis using the example of unexposed cell control (**a**), rutile nanorods (**b**) und CMC-functionalized rutil nanorods (**c**).

Reactive oxygen species (ROS) FACS analysis

The intracellular generation of ROS in NR8383 cells upon incubation with the different TiO_2 particles was measured by the DCF assay. The gating strategy is illustrated in Figure S2 using a 3% H_2O_2 solution as a high ROS level inducer. Dead cells were excluded by gating PI positive cells, and ROS production was determined by gating the DCF positive fraction substracting the number of dead cells. Data are given as mean±SD (n=3) and percentage of control (100%, H_2O_2-treated cells).

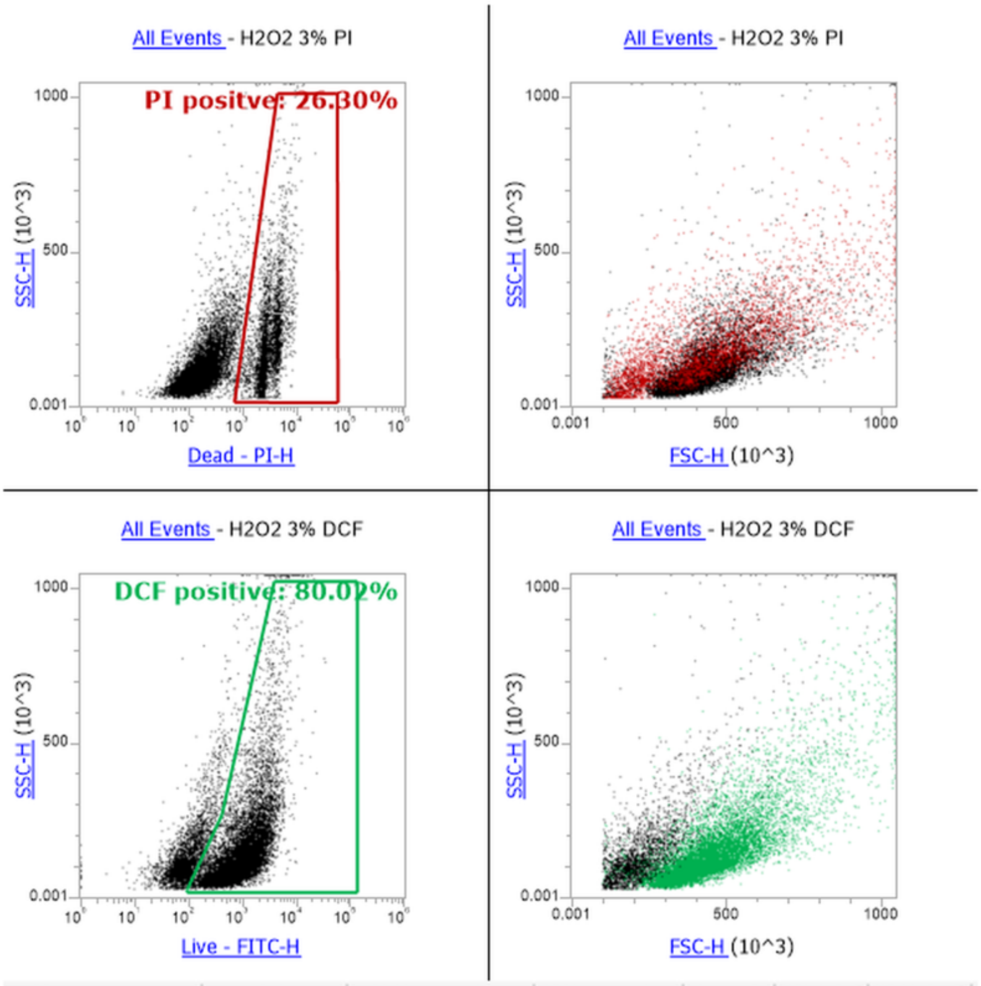

Name	Gate	X Parameter	Y Parameter	Count	%Total	%Gated
PI positive	PI positive	Dead-PI - BL2-H	SSC - SSC-H	3,945	26.30	26.30
DCF positive	DCF positive	Live-FITC - BL1-H	SSC - SSC-H	12,003	80.02	80.02

Figure S2: Gating strategy for the detection of generated ROS in NR8383 cells by flow cytometric analysis using the example of cells exposed to 3% H_2O_2 for 2 h.

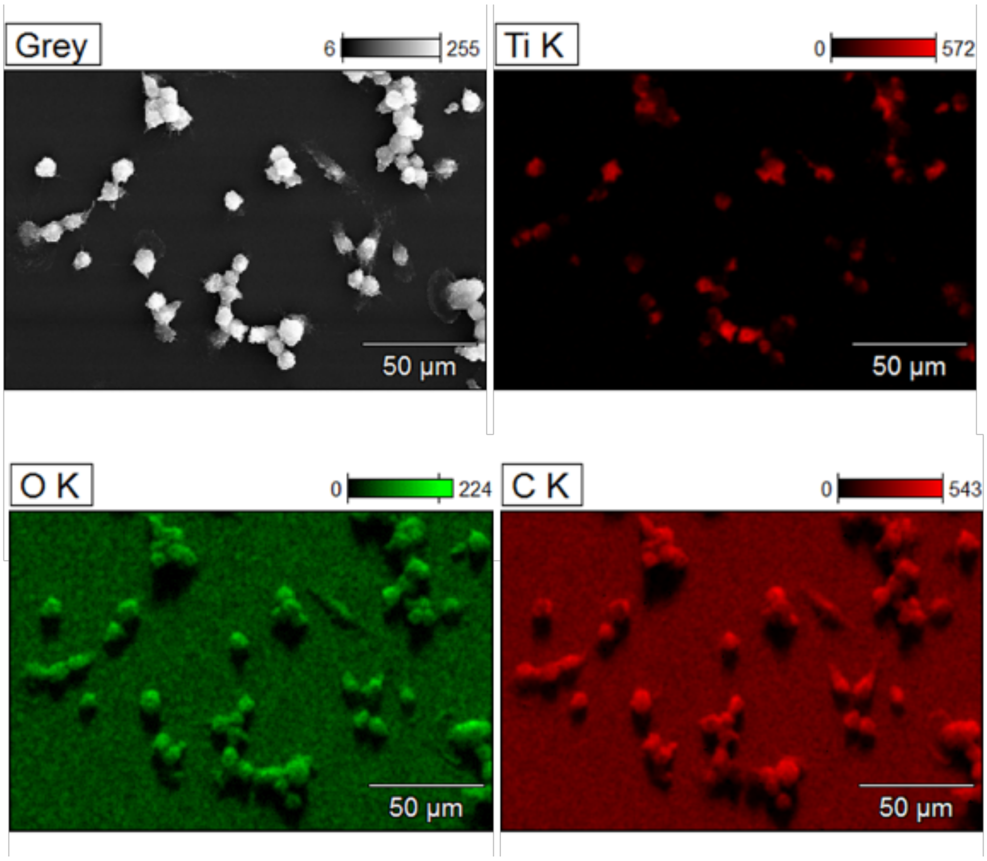

Figure S3: EDS elemental mapping of NR8383 macrophages after incubation with CMC-functionalized anatase microspheres for 24 h.

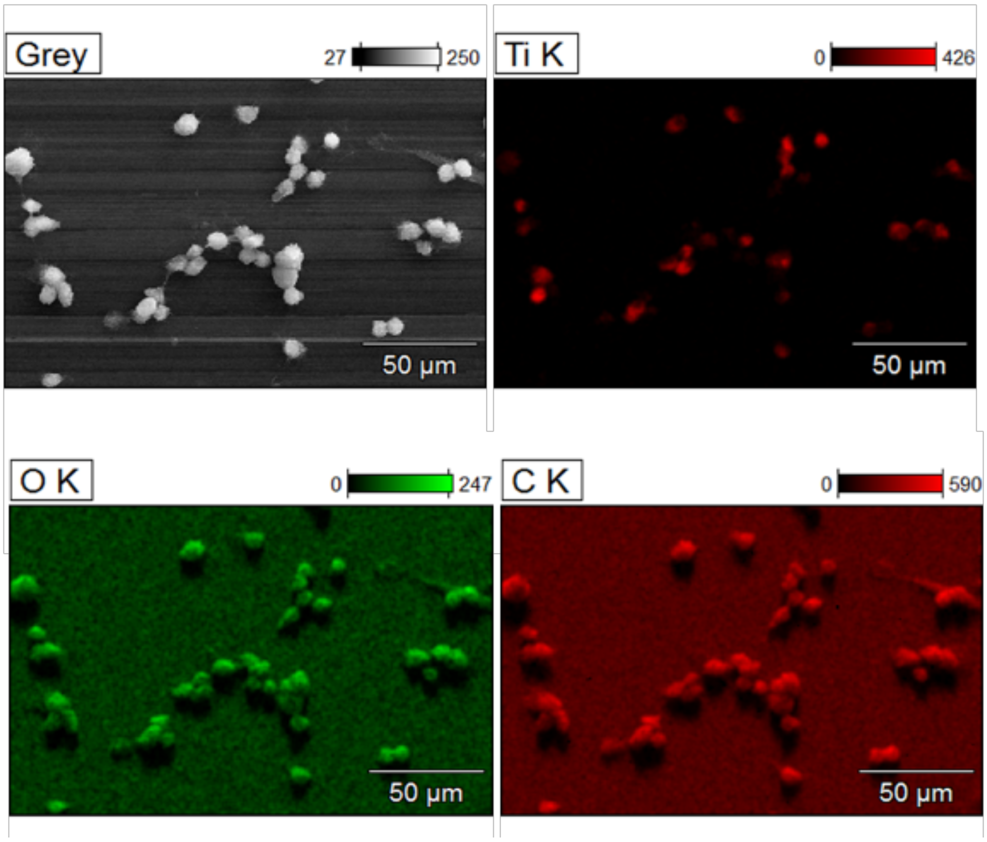

Figure S4: EDS elemental mapping of NR8383 macrophages after incubation with unfunctionalized anatase nanospheres for 24 h.

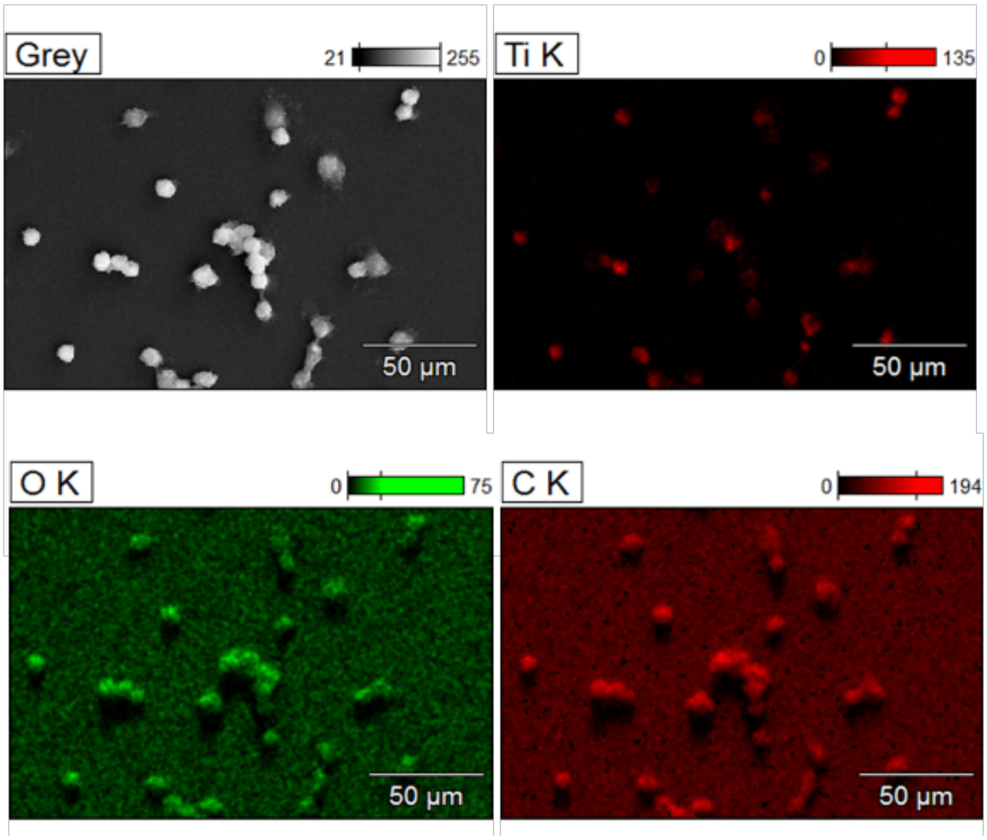

Figure S5: EDS elemental mapping of NR8383 macrophages after incubation with CMC-functionalized anatase nanospheres for 24 h.

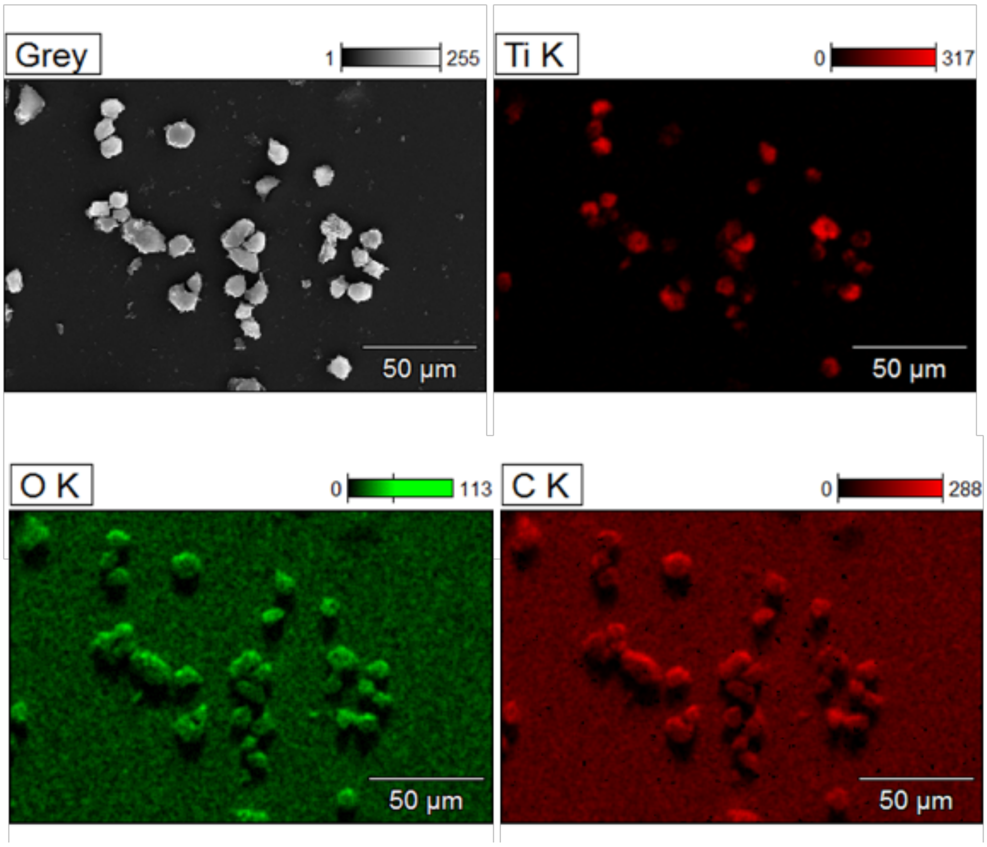

Figure S6: EDS elemental mapping of NR8383 macrophages after incubation with unfunctionalized rutile microspheres for 24 h.

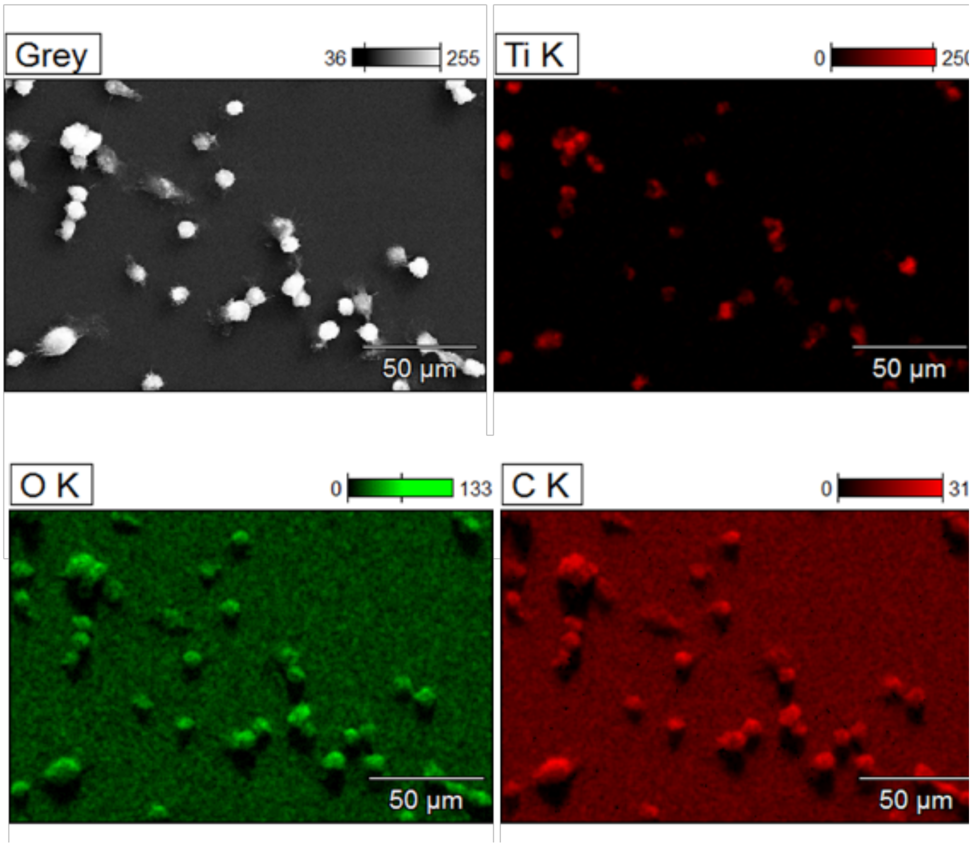

Figure S7: EDS elemental mapping of NR8383 macrophages after incubation with CMC-functionalized rutile microspheres for 24 h.

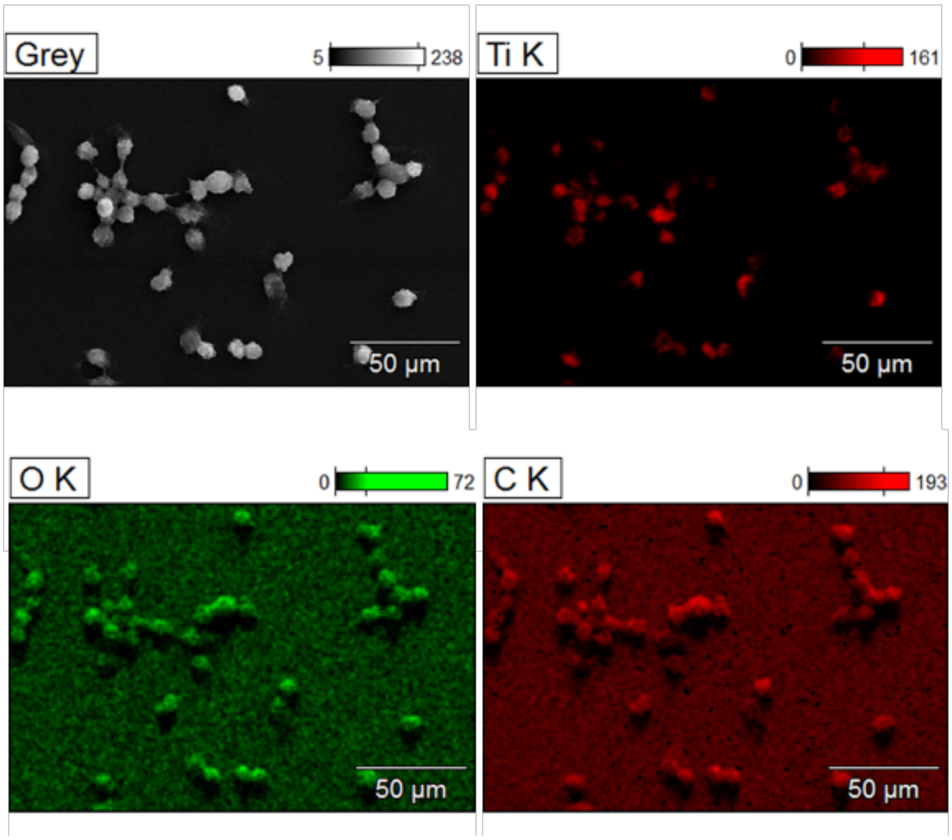

Figure S8: EDS elemental mapping of NR8383 macrophages after incubation with unfunctionalized rutile submicrorods for 24 h.

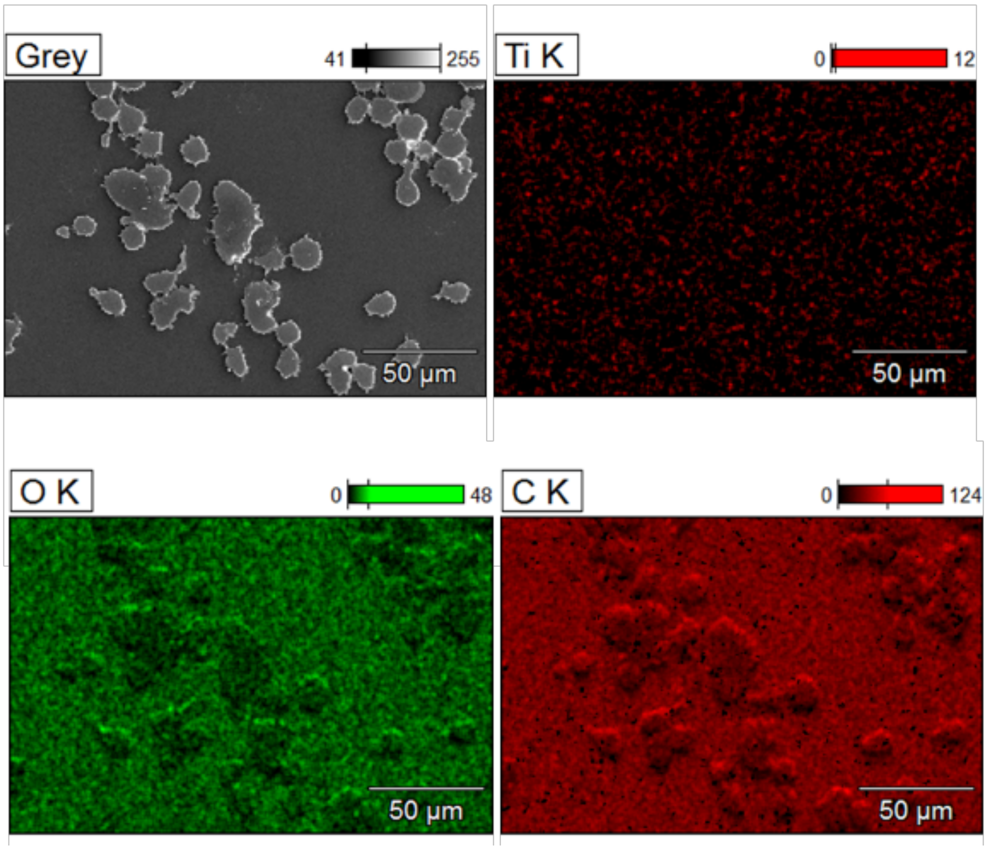

Figure S9: EDS elemental mapping of NR8383 macrophages after incubation with CMC-functionalized rutile submicrorods for 24 h.

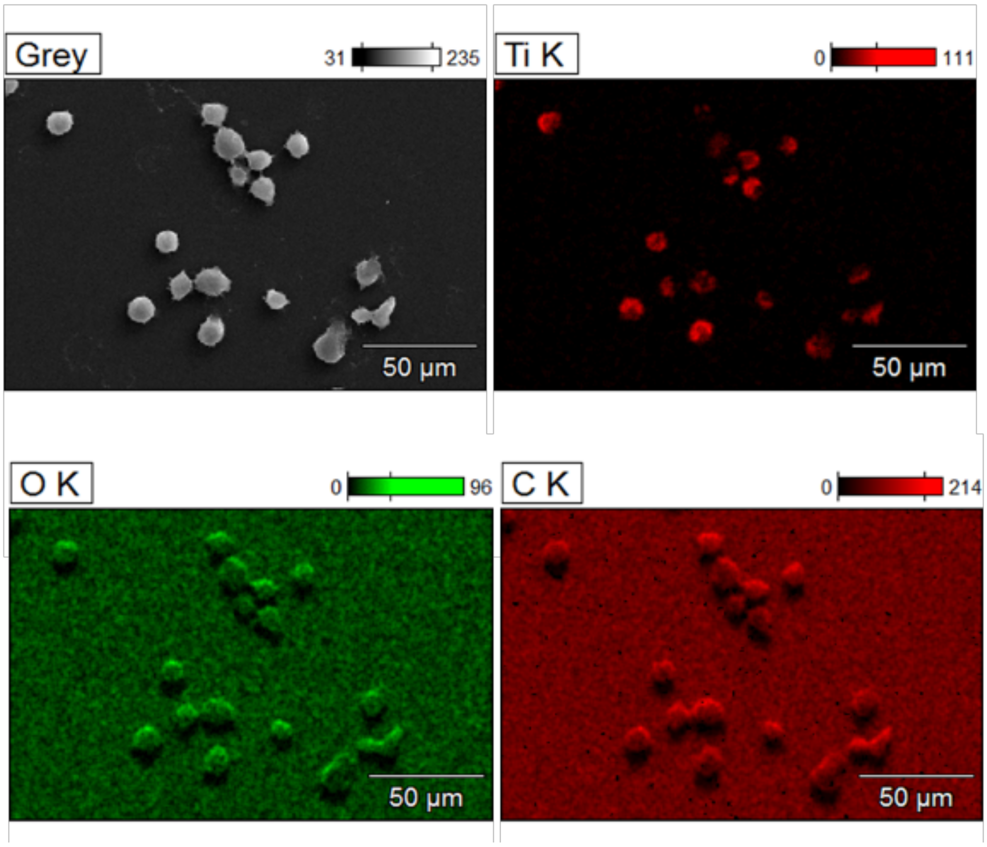

Figure S10: EDS elemental mapping of NR8383 macrophages after incubation with unfunctionalized rutile nanorods for 24 h.

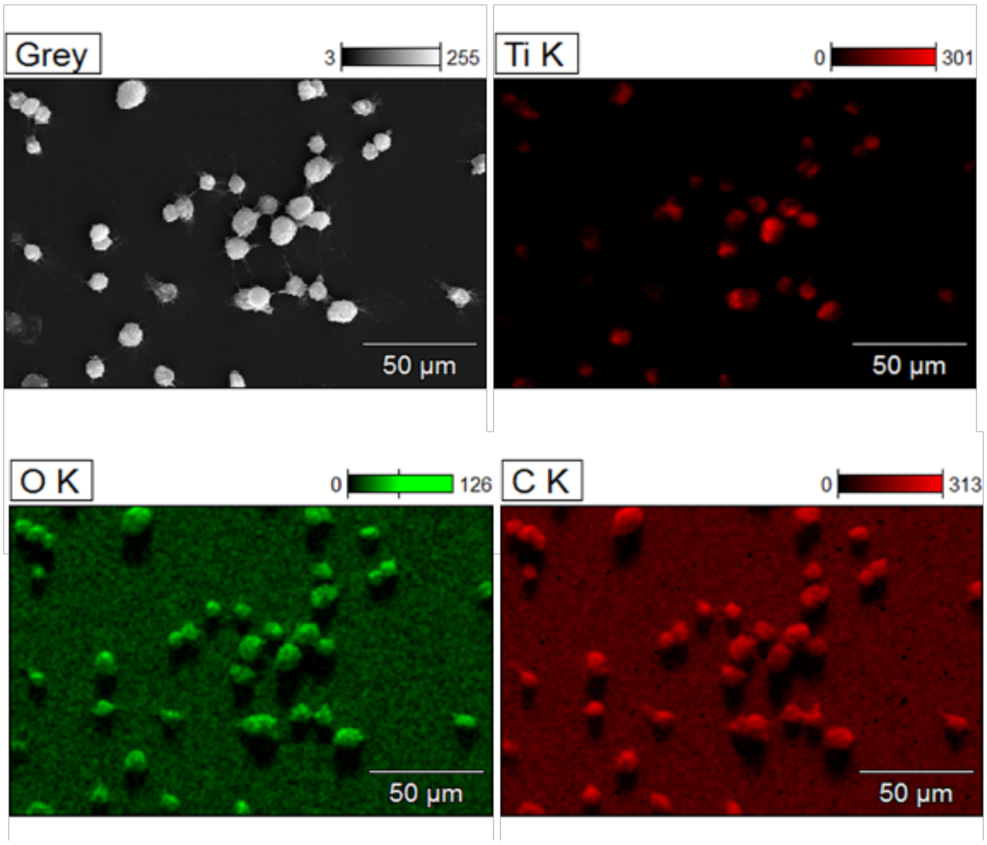

Figure S11: EDS elemental mapping of NR8383 macrophages after incubation with CMC-functionalized rutile nanorods for 24 h.

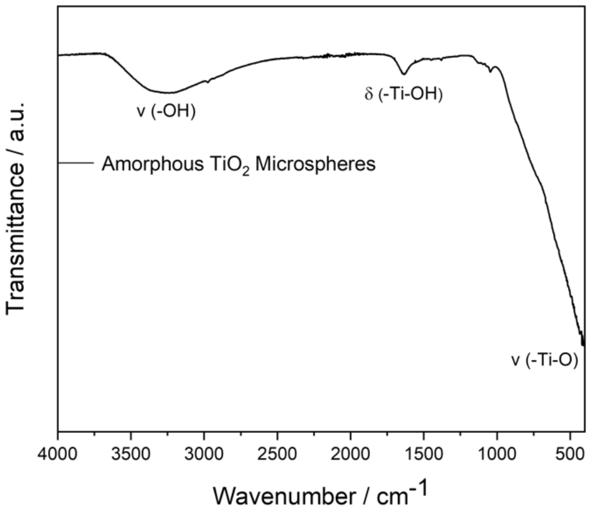

Figure S12: IR spectrum of amorphous TiO_2 microspheres.

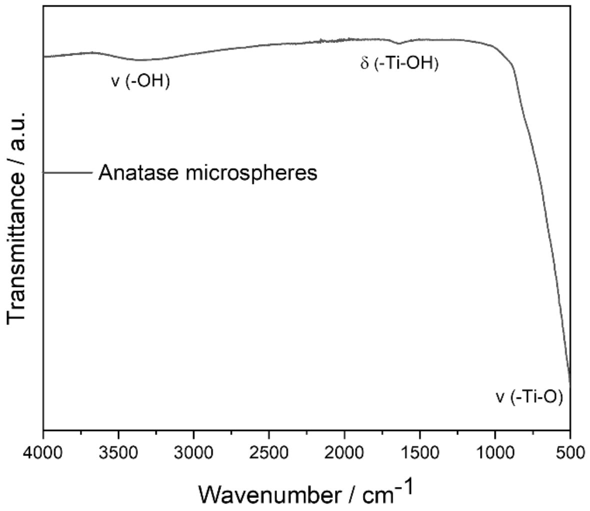

Figure S13: IR spectrum of anatase microspheres.

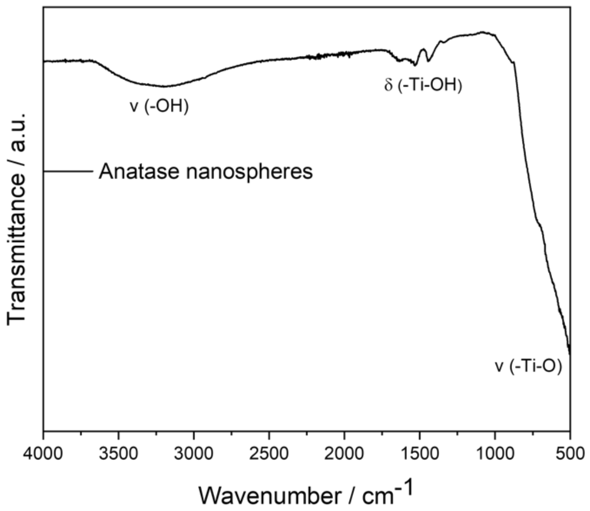

Figure S14: IR spectrum of anatase nanospheres.

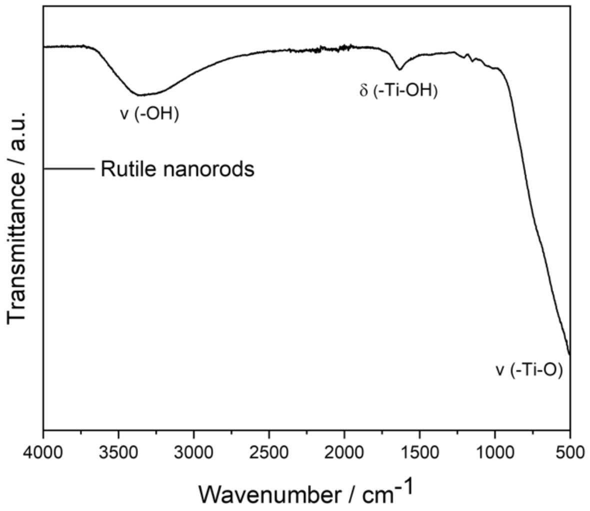

Figure S15: IR spectrum of rutile nanorods.

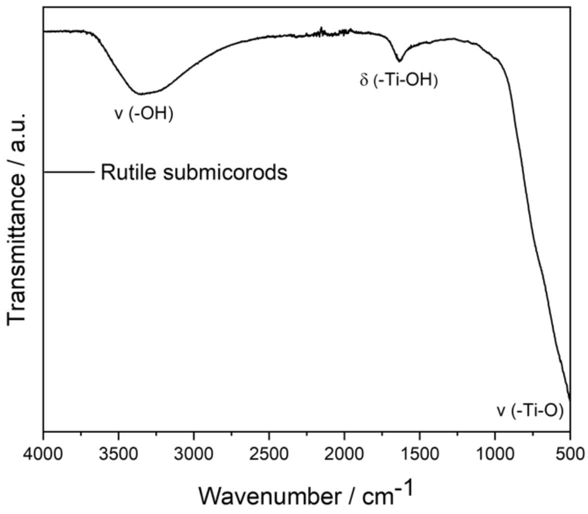

Figure S16: IR spectrum of rutile submicrorods.

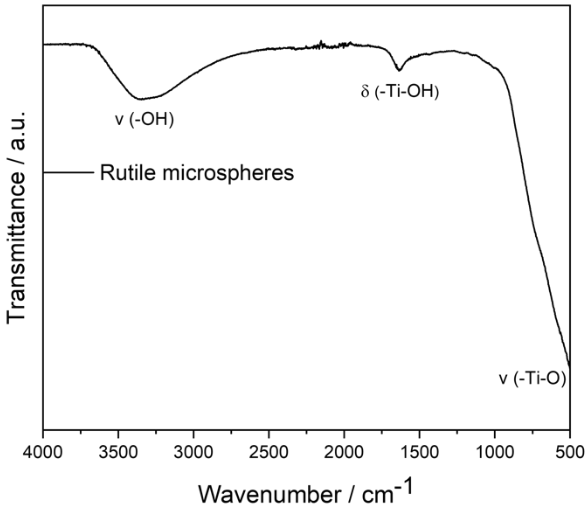

Figure S17: IR spectrum of rutile microrods.

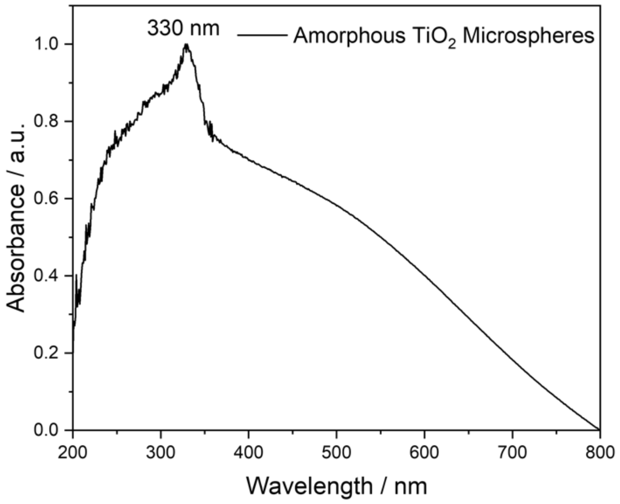

Figure S18: UV/vis spectrum of amorphous TiO_2 microspheres.

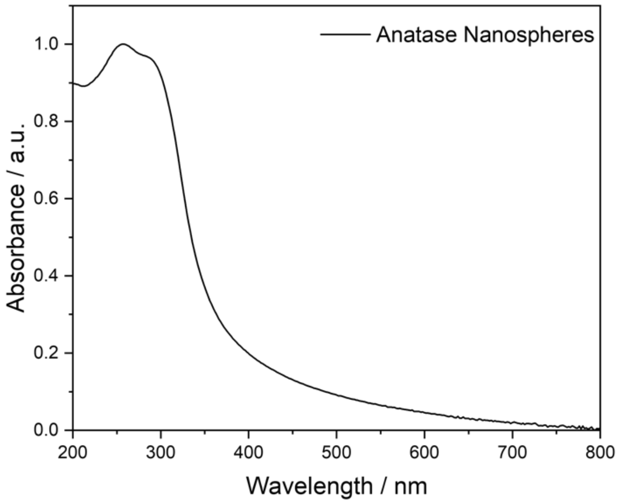

Figure S19: UV/vis spectrum of anatase nanospheres.

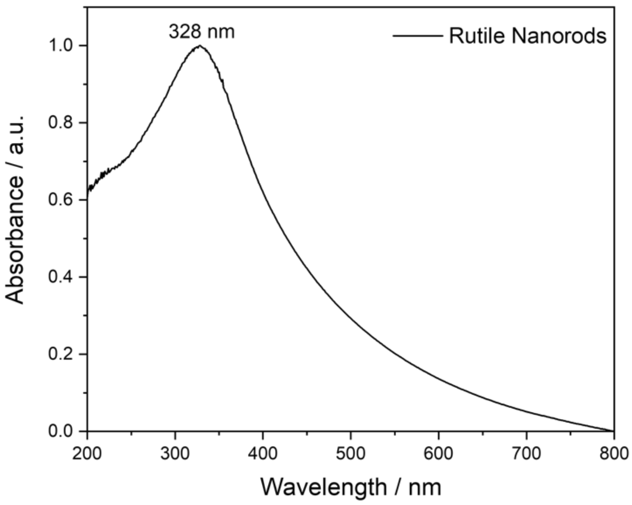

Figure S20: UV/vis spectrum of rutile nanorods.

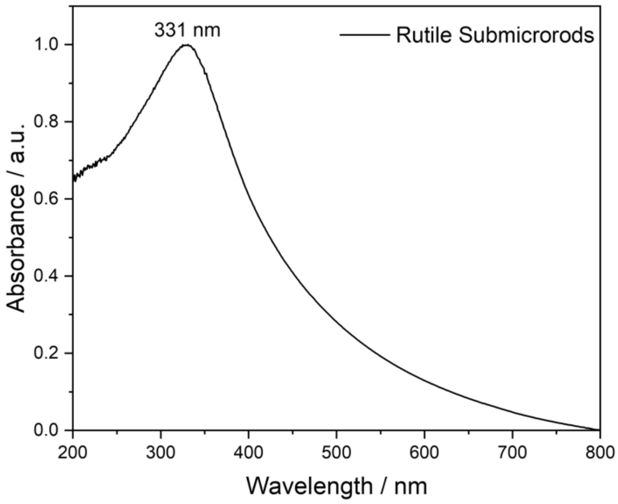

Figure S21: UV/vis spectrum of rutile submicrorods.
